# Notes on the Recent History of Neuroscience in Africa

**DOI:** 10.3389/fnana.2017.00096

**Published:** 2017-11-07

**Authors:** Vivienne A. Russell

**Affiliations:** ^1^Department of Human Biology, Faculty of Health Sciences, University of Cape Town, Observatory, Cape Town, South Africa; ^2^School of Laboratory Medicine and Medical Sciences, College of Health Sciences, University of KwaZulu-Natal, Durban, South Africa

**Keywords:** brain, nervous system, neuroanatomy, neurosurgery, neurology, neuropsychiatry, neurophysiology

## Abstract

Neuroscience began with neuroanatomy and neurosurgery in Egypt more than 5000 years ago. Knowledge grew over time and specialized neurosurgery centers were established in north Africa in the eleventh century. However, it was not until the twentieth century that neuroscience research became established in sub-Saharan Africa. In most African countries, clinical research focused on understanding the rationale and improving treatment of epilepsy, infections, nutritional neuropathies, stroke and tumors. Significant advances were made. In the twenty-first century, African knowledge expanded to include all branches of neuroscience, contributing to genetic, biochemical and inflammatory determinants of brain disorders. A major focus of basic neuroscience research has been, and is, investigation of plant extracts, drugs and stress in animal models, providing insight and identifying potential novel therapies. A significant event in the history of African neuroscience was the founding of the Society of Neuroscientists of Africa (SONA) in 1993. The International Brain Research Organization (IBRO) supported SONA conferences, as well as workshops and neuroscience training schools in Africa. Thanks to their investment, as well as that of funding agencies, such as the National Institutes of Health (NIH), International Society for Neurochemistry (ISN), World Federation of Neurosurgical Societies (WFNS), World Federation of Neurology (WFN) and the International League Against Epilepsy (ILAE), neuroscience research is well-established in Africa today. However, in order to continue to develop, African neuroscience needs continued international support and African neuroscientists need to engage in policy and decision-making to persuade governments to fund studies that address the unique regional needs in Africa.

## Introduction

The earliest evidence of neuroscience research dates back more than 5000 years. Egyptian embalmers were the first to obtain knowledge of human anatomy through the mummification process. However, they had very little regard for the brain and did not try to preserve it (Cappabianca et al., 2007; Elhadi et al., 2012). The oldest record of neuroscience research, the Edwin Smith Papyrus, is believed to have been written around 2620 BC by the Egyptian physician and architect, Imhotep who was also a high priest of the sun god Ra (Elhadi et al., 2012). The Edwin Smith Papyrus reports traction as the first recorded neurosurgical procedure, used to reverse a paralyzing spinal injury in an Egyptian leader around 3000 BC (Filler, 2007). There is also evidence of early knowledge of the association between cerebral lesions and loss of movement on the contralateral side of the body, as well as fractures of the cervical spine being associated with neck rigidity, limb paralysis and conjugate eye deviation (El-Gindi, 2001). Trepanation, the process of creating a burr hole in the skull to access the brain, was widely used to relieve pressure after head injury, and is used to this day in the diagnosis and treatment of patients with traumatic brain injury in many African countries, especially those where modern techniques such as computerized tomography scans are not available (Eaton et al., 2017).

For more than 3000 years, Egypt was the center of knowledge of human brain structure and function. Early physicians studied neuroanatomy at the medical school in Memphis (Elhadi et al., 2012). However, religious conflicts restricted the study of human anatomy and myths replaced scientific research for centuries (Elhadi et al., 2012). It was not until Alexander the Great conquered Egypt in 332 BC and founded the city of Alexandria that significant advances were made (Elhadi et al., 2012). His conquest of the Persian Empire opened communication and promoted the exchange of knowledge across a world of previously hostile nations (Elhadi et al., 2012). A revolution in the study of functional anatomy followed. Great men such as Galen, Herophilus, Erasistratus and Rufus were prominent physicians who studied at the medical school in Alexandria (Elhadi et al., 2012). The Library of Alexandria became known for its impressive collection of recorded knowledge which attracted philosophers, scientists and teachers to Alexandria to study, debate and conduct scientific investigations (Elhadi et al., 2012). Egyptians were known for their skill in the practice of medicine. Their lively spirit of enquiry lead to discoveries in what was regarded as the science or philosophy of the day (Elhadi et al., 2012). These early neuroscientists carefully documented their detailed systematic dissections of human and animal nervous systems, tracing nerves to the brain, debating the function of nerve fibers originating in the brain stem and spinal cord, differentiating between sensory and motor nerves and attributing motor function to these nerves (Elhadi et al., 2012). Alexandria continued to be the center of neuroscience research until 30 BC when the Romans conquered Egypt and laws were passed that prohibited human dissections thereby preventing further progress in acquiring knowledge of the anatomy and physiology of the human nervous system for the next 1500 years (Elhadi et al., 2012).

## Progress in the Twentieth and Twenty-First Century

Close proximity to Middle East and European training schools encouraged north African neuroscientists to further their studies and thereby contribute to the advancement of neuroanatomy and neurosurgery in these countries (El-Fiki, 2010). Knowledge grew over time and specialized neurosurgery centers were established in north Africa in the eleventh century (El Khamlichi, 1998). Modern neurosurgery was subsequently introduced to many countries in Africa in the twentieth century, mainly as a result of colonization by France and Britain (El Khamlichi, 1998). Departments of Neurosurgery and Neurology were established in African cities but these were initially staffed by foreigners (El Khamlichi, 1998). Unfortunately, the first African generation of neuroscientists who were trained in foreign countries did not stay in Africa (El-Fiki, 2010). They were unhappy with the lack of equipment and failure of existing equipment, largely due to poor maintenance, as well as the poor working conditions and absence of basic research facilities (El-Fiki, 2010). This situation began to change as some African neuroscientists returned to their home countries during the twentieth century and the African diaspora began to contribute significantly to the development of neuroscience research in Africa. Strong ties to foreign Universities helped to establish neuroscience research which developed rapidly in the latter part of the twentieth century. Spinal surgery, for example, advanced from decompression to spinal reconstruction and internal stabilization as a result of the introduction of computerized tomography and magnetic resonance imaging, and basic neuroscience research became established in many African countries (Loots et al., 1975; Shanley et al., 1975; Bengelloun et al., 1976; Wangai et al., 1978; Hattingh et al., 1979; Kimani and Mungai, 1983; Anderson et al., 1985; Nurse et al., 1985; Lakhdar-Ghazal et al., 1986; Bennis and Versaux-Botteri, 1995; McDonnell, 2004).

In most countries in Africa, clinical neuroscience research focused on understanding the rationale and improving treatment of neurological disorders, including epilepsy, infections (predominantly cerebral malaria, meningitis, encephalitis, poliomyelitis, leprosy, tetanus), nutritional neuropathies, stroke, tumors, motor disorders and the adverse effects of snake venom (Lambo, 1956; Osuntokun et al., 1968; Dada et al., 1969; Kramer et al., 1971; Wangai et al., 1978; Anderson et al., 1985; Tazir and Geronimi, 1990; Bhigjee et al., 1993; Vallat et al., 1993; Hentati et al., 1994; Ogunniyi, 2010). Significant advances were made. In the twenty-first century, African knowledge expanded rapidly to embrace all branches of neuroscience. Research covered genetic, biochemical and inflammatory determinants of brain disorders as well as basic neuroscience research on local fauna and animal models of brain disorders. An increasing focus of basic neuroscience research in Africa was in the field of neuropharmacognosy. Many countries have been, and still are, investigating the disease-modifying benefits of administering plant extracts to experimental animals (Ojewole and Amabeoku, 2006; Bum et al., 2009; Oboh et al., 2012; Eduviere et al., 2015; Akinrinmade et al., 2017; Manouze et al., 2017; Ngoupaye et al., 2017). African neuroscientists have the advantage of access to unique ecosystems of high biodiversity as well as critical knowledge of traditional medicine which has great potential to lead to the discovery of novel bioactive compounds (Karikari and Aleksic, 2015). African neuroscientists are also beginning to use invertebrate model organisms such as *Drosophila melanogaster* as powerful low-cost alternatives to animal models for testing natural products which will strengthen their ability to identify bioactive plant extracts with therapeutic potential (Karikari and Aleksic, 2015; Akinyemi et al., 2017).

**Figure 1:**
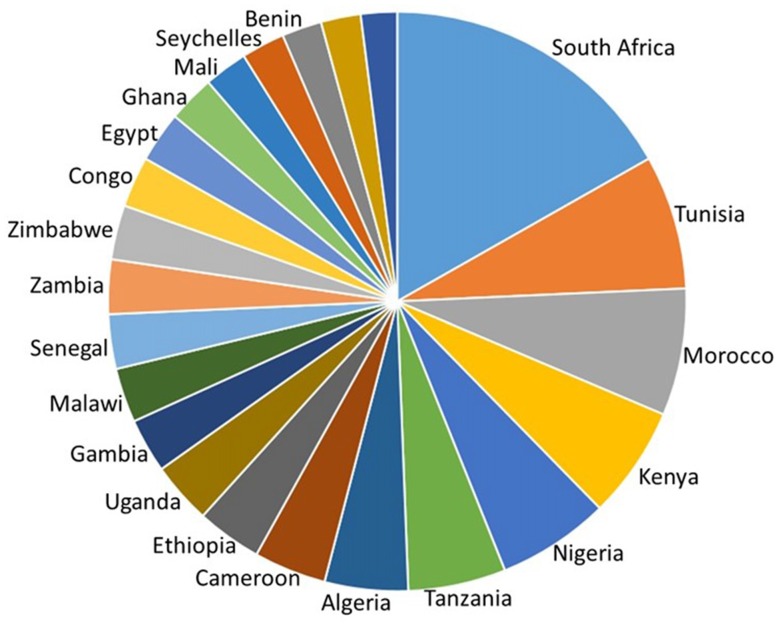
H-index of countries in Africa calculated as the number of neuroscience publications that have each been cited at least that number of times in other articles. South Africa, Tunisia, Morocco, Kenya, Nigeria, Tanzania, Algeria and Cameroon have the highest country H index. Adapted from SCImago (2007) http://www.scimagojr.com/countryrank.php?area=2800&region=Africa&order=h&ord=desc and https://knoema.com/GII2017/global-innovation-index-2016.

It is impossible to do adequate justice to all of the neuroscience research that has been carried out in Africa. This mini-review will therefore trace a few of the major trends based on a PubMed search of neuro-related publications by African neuroscientists in the eight African countries listed as having an H index of 20 or greater, in the SCImago Journal & Country Rank Report (2016)^1^. These countries have at least 20 neuroscience publications that have earned at least 20 citations each. They are, from north to south; Tunisia, Algeria, Morocco, Nigeria, Cameroon, Kenya, Tanzania and South Africa (Figure 1).

## North Africa

**Figure 2:**
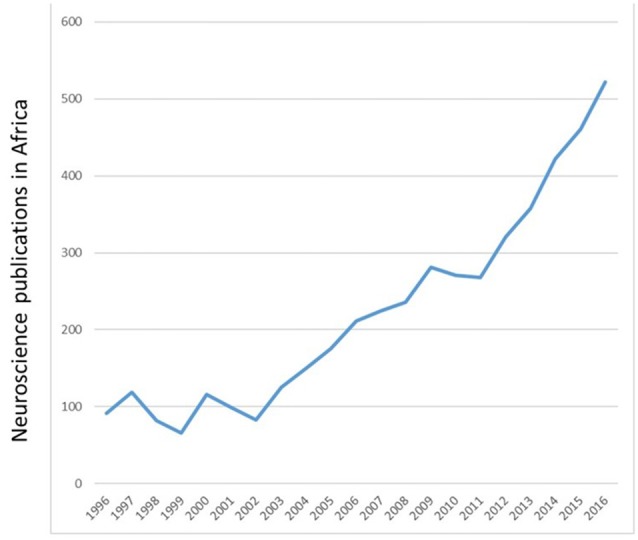
Number of Neuroscience publications *per annum* from 1996 to 2016. Adapted from SCImago (2007) http://www.scimagojr.com/worldreport.php?area=2800&w=Africa.

In the twentieth and twenty-first centuries, neuroscience research in Tunisia and Algeria involved mostly clinical studies on neurogenetics and movement disorders (Tazir and Geronimi, 1990; Hentati et al., 1994; Gouider-Khouja et al., 2000; Younes-Mhenni et al., 2007). Clinical research in Morocco included stroke, Alzheimer’s disease, addiction and fMRI studies of brain plasticity, to mention a few (El Kadmiri et al., 2014; Souirti et al., 2014; Mohamed and Kissani, 2015; Chtaou et al., 2016; Zarrouq et al., 2016; Boujraf et al., 2017). Basic neuroscience research was introduced to Morocco in the 1970s with the study of behavioral consequences of brain lesions and nutritional deficiency in rats (Bengelloun et al., 1976). In the 1980s and more recently, the focus of researchers across the country expanded to include not only brain lesions and malnutrition but also stress, drugs of abuse, neurotoxins, sensory systems and biological rhythms in laboratory animals and local fauna (Lakhdar-Ghazal et al., 1986; Bennis and Versaux-Botteri, 1995; Sansar et al., 2012; Said et al., 2015). Recent years have seen a marked increase in the number of publications by African neuroscientists (Figure 2).

## Sub-Saharan Africa

The earliest knowledge of neurological disorders in sub-Saharan Africa is attributed to Yoruba Traditional healers in Nigeria, in the seventeenth century (Ogunniyi, 2010). However, it was only in the middle of the 20th century that neuroscience research began to flourish in sub-Saharan Africa. In the 1950s, the first black African neuropsychiatrist introduced a community–based system of treatment of psychiatric patients in Nigeria, a form of treatment that remains relevant to this day (Lambo, 1956; Ogunniyi, 2010). Several articles describing disorders of the nervous system were published in the 1960s (Odeku, 1965; Osuntokun, 1968; Dada et al., 1969; Osuntokun et al., 1969b; Okubadejo et al., 2006; Ogunniyi et al., 2015). In 1969, the tropical ataxic neuropathy, konzo, was attributed to the cyanogenic glycosides present in cassava (Osuntokun et al., 1969a). In the 1970s and early 1980s, neuroepidemiological studies dominated tropical neurology (Osuntokun, 1978; Ogunniyi, 2010). More recently, research has focused on neuroprotective properties of indigenous plant extracts, neurotoxicity of environmental factors, neurogenomics, stroke, epilepsy and neurodegenerative diseases, including Parkinson’s disease and dementia, amongst others (Okubadejo et al., 2006; Akinyemi et al., 2014, 2016; Lekoubou et al., 2014; Mkenda et al., 2016; Ekong et al., 2017; Folarin et al., 2017; Ilesanmi et al., 2017; Ojagbemi et al., 2017).

Neuropharmacognosy is an important component of basic neuroscience research in many countries in Africa, where native African traditional medicines are scientifically analyzed with a view to validating their benefits and thereby offer potential novel therapies (Amos et al., 2001; Ojewole and Amabeoku, 2006; Bum et al., 2009; Bisong et al., 2010; Ishola et al., 2013; Ogunniyi et al., 2015; Qulu et al., 2016; Adebesin et al., 2017; Elufioye et al., 2017; Ngoupaye et al., 2017).

In Kenya, the earliest studies on the nervous system were largely descriptive and focused on brain size rather than function (Vint, 1934). As in many African countries that were colonized by Britain, the development of modern neurosurgical procedures in Kenya occurred in the late 1940s, as a result of the two world wars (Qureshi and Oluoch-Olunya, 2010). The last quarter of the twentieth century saw neurosurgery develop to its present level, with Kenyan neurosurgeons engaged in both practice and research (Qureshi and Oluoch-Olunya, 2010). The earliest reports on basic neuroscience research in Kenya appeared in the late 1970s and early 1980s (Wangai et al., 1978; Kimani and Mungai, 1983; Anderson et al., 1985). More recently, research has focused on morphine-induced aggression and antinociceptive effects in the naked mole-rat, antinociceptive and anti-inflammatory effects of plant extracts in mice as well as the mechanism of action of cathinone (active ingredient of khat, *Catha edulis*) an addictive psychostimulant grown in abundance in Kenya and impairment of executive function in children with malaria, to name a few (Kanui and Hole, 1990; Kanui et al., 1993; Patel, 2000, 2015; Kariuki et al., 2013, 2014; Jørgensen et al., 2016; Kimani et al., 2016). In Tanzania and Cameroon, a dominant focus of neuroscience research has been epilepsy, infectious diseases, stroke and assessment of the potential therapeutic value of indigenous plant extracts (Matuja et al., 2001; Njamnshi et al., 2006, 2009, 2012; Bum et al., 2009; Levira et al., 2017; Ngoupaye et al., 2017).

The earliest publication on the nervous system, from South Africa, examined the embryonic history of the segmented mesoderm and neural tube (Dart, 1924). Basic and clinical neuroscience research emerged in the 1950s and 1960s with the neurophysiology of the spinal cord and research on *Cannabis sativa* (Ames, 1958; Holemans et al., 1966). In the 1970s research expanded to include neurological disorders, including porphyria, psychiatric disorders, nociception and thermoregulation in different animal species (Kramer et al., 1971; Loots et al., 1975; Shanley et al., 1975; Woolf et al., 1977; Hattingh et al., 1979). In the 1980s and more recently, in collaboration with other countries in Africa, attempts were made to understand the success of traditional healers in the treatment of patients with psychiatric disorders (Wessels, 1985; Gureje et al., 2015). Research has expanded in all branches of neuroscience to include neuroimaging studies of e.g., children with fetal alcohol spectrum disorder which is highly prevalent in the Western Cape region of South Africa, as well as neurodevelopmental outcomes of children with tuberculous meningitis and hydrocephalus, which is also highly concentrated in this region, as well as neuropsychiatric genomics, psychiatric disorders such as post-traumatic stress disorder, HIV-associated neurocognitive disorders, schizophrenia, bipolar disorder, drug addiction, the neuropsychology of emotional experience, neurology, neurosurgery, functional neuroanatomy including neurogenesis and understanding neural disturbances in animal models of brain disorders, to name a few (Howells et al., 2016; Qulu et al., 2016; Rohlwink et al., 2016; Sterley et al., 2016; van Wyk et al., 2016; Dallé et al., 2017; Kilian et al., 2017; Mazengenya et al., 2017; Panksepp et al., 2017; Uys et al., 2017; Womersley et al., 2017; du Plessis et al., 2017).

This mini-review cannot provide a comprehensive overview but seeks to highlight some of the research carried out in those countries in Africa that have achieved recognition in terms of the number of neuroscience publications that have been cited by other researchers, according to the SCImago Journal & Country Rank Report (2016)^2^. However, there is no mention of all the excellent research that has been, and is being carried out in countries not listed here, for example, research on konzo in the Democratic Republic of Congo, nodding syndrome and cerebral malaria in Uganda, to mention a few (Bumoko et al., 2015; Bangirana et al., 2016; Idro et al., 2016).

## Training in Neuroscience in Africa

Over the years, science-based non-profit organizations such as the International Brain Research Organization (IBRO) have supported African neuroscientists and helped to build scientific capacity for sustainable education and research by sponsoring training courses and workshops across Africa. Training programmes in Neurosurgery, Neurology and basic Neuroscience, supported by, the World Federation of Neurosurgical Societies (WFNS), the World Federation of Neurology (WFN), the Society of French Speaking Neurosurgeons (SNCLF) the International League Against Epilepsy (ILAE), the National Institutes of Health (NIH), IBRO and the International Society of Neurochemistry (ISN), amongst others, have led to the production of many generations of neuroscientists in Africa. IBRO and ISN, in particular, have engaged in basic neuroscience with a strong translational approach to brain disorders in Africa. IBRO African Centres for Advanced Training in Neuroscience have been established in Morocco and South Africa. WFNS-recognized Centres of Excellence for training neurosurgeons have been established in Nigeria, Ivory Coast, Senegal, Kenya, Zimbabwe and South Africa (Dechambenoit, 2010).

A significant event in the history of African neuroscience is the founding of the Society of Neuroscientists of Africa (SONA) by James Kimani in 1993. IBRO generously supported SONA conferences, as well as several workshops and neuroscience training schools in more than 14 African countries. Thanks to their investment, as well as that of the other funding agencies, neuroscience research is well-established in Africa. However, in order to continue to develop, African neuroscience needs continued international support and African neuroscientists need to engage in policy and decision-making to persuade governments to fund studies of unique regional needs in Africa (Bentivoglio et al., 2014). Basic neuroscience is not a luxury but a means to address the challenges of specific needs in disease-endemic regions (addiction, toxic pollutants, infections) by investigating the pathogenic mechanisms (in neglected tropical diseases and conditions) and taking advantage of unique models (the brain and behavior of African fauna) and unique populations (children with fetal alcohol spectrum disorder) to better understand adaptations to environmental conditions and susceptibility to disease (Bentivoglio et al., 2014). For instance, a better understanding of the nervous system of the mosquito or the tsetse fly can lead to better methods to control these disease vectors (Kristensson et al., 2010; Sparks and Dickens, 2016). African problems need African solutions, goal-directed basic neuroscience research is needed in Africa where there is a dire need to combat regionally-specific devastating brain disorders (Bentivoglio et al., 2014).

## Author Contributions

VAR wrote the mini-review.

## Conflict of Interest Statement

The author declares that the research was conducted in the absence of any commercial or financial relationships that could be construed as a potential conflict of interest.

## Footnotes

^1^SCImago (2007) http://www.scimagojr.com/worldreport.php?area=2800

^2^http://www.scimagojr.com/worldreport.php?area=2800
